# The peritumoral hypointense rim around hepatocellular carcinoma on T2*-weighted magnetic resonance imaging: radiologic–pathologic correlation

**DOI:** 10.1186/s12957-021-02152-2

**Published:** 2021-02-06

**Authors:** Yoshinori Tsukahara, Yukinori Okajima, Akira Yamada, Masanobu Momose, Takeshi Uehara, Akira Shimizu, Yuji Soejima, Yasunari Fujinaga

**Affiliations:** 1 Department of Radiology, Shinshu University School of Medicine, 3-1-1 Asahi, Matsumoto, Nagano, 390-8621 Japan; 2 Department of Laboratory Medicine, Shinshu University School of Medicine, 3-1-1 Asahi, Matsumoto, Nagano, 390-8621 Japan; 3 Department of Surgery, Shinshu University School of Medicine, 3-1-1 Asahi, Matsumoto, Nagano, 390-8621 Japan

**Keywords:** Hepatocellular carcinoma, Magnetic resonance imaging, Iron, Prussian blue reaction

## Abstract

**Background:**

A peritumoral hypointense rim (PTHR) is sometimes observed around hepatocellular carcinoma (HCC) on T2*-weighted images (T2*WIs). We aimed to investigate the association between the PTHR and histopathologic findings on T2*WIs.

**Methods:**

We assessed the presence of a PTHR on T2*WIs in 39 pathologically proven HCCs from April 2012 to December 2013. Prussian blue staining was performed, and iron deposition was evaluated by semiquantitative and quantitative methods. Optical density was used in the quantitative methods. The associations between a PTHR on T2*WI and histopathologic peritumoral or background liver iron deposition were analyzed.

**Results:**

A PTHR on T2*WI was observed in 23 of 39 (59%) HCCs. There was no significant difference in the histopathologic fibrous capsule findings (*P* = 0.394). In the semiquantitative methods, both peritumoral and background liver iron deposition grade were significantly higher in HCCs with a PTHR compared with HCCs without a PTHR (*P* < 0.001). The mean optical density in HCCs with a PTHR was significantly higher compared with HCCs without a PTHR, in the quantitative peritumoral (42,244.1 ± 20,854.9 vs. 18,739.1 ± 12,258.7, respectively; *P* < 0.001) and background liver iron deposition analyses (35,554.7 ± 19,854.8 vs. 17,292.4 ± 11,605.8, respectively; *P* < 0.001). Tumor size (*P* = 0.005), etiology (*P* = 0.001), and degree of fibrosis (*P* = 0.042) were significantly associated with the presence of a PTHR.

**Conclusions:**

A PTHR in HCCs on T2*WIs was strongly associated with peritumoral iron deposition in the iron-deposited background liver but not with the fibrous capsule.

## Background

Magnetic resonance (MR) imaging is a useful modality for diagnosing hepatocellular carcinoma (HCC). In particular, dynamic contrast-enhanced (DCE) MR imaging is a very useful technique for detecting the characteristic findings of HCC, such as arterial phase enhancement, washout appearance, and enhancing capsule appearance [1–3]. However, the administration of gadolinium contrast agent to patients with renal failure and previous hypersensitivity reactions is contraindicated [4, 5].

A fibrous capsule is a characteristic histopathologic feature of HCC. In MR imaging, the fibrous capsule appears as a hypointense band around the tumor that can be detected in nonenhanced T1-weighted images (T1WIs) and T2-weighted images (T2WIs) without a contrast agent [2, 6]. This feature was adopted as an ancillary feature favoring HCC in the Liver Imaging Reporting and Data System (LI-RADS), which is a comprehensive system for standardizing liver imaging diagnosis [7].

Chen et al. reported that a peritumoral hypointense rim (PTHR) in HCC on T2*WIs or susceptibility-weighted images (SWIs) was superior to T1WIs and T2WIs for assessing capsule appearance [8]. Therefore, PTHR may be a useful feature for diagnosing HCC without administering a contrast agent. However, the report did not show an association with a histopathologic fibrous capsule, and the report’s findings have not been analyzed in detail.

T2*-weighted images (T2*WIs) are sensitive to magnetic susceptibility and magnetic body components, such as iron deposits and microbleeds in tissues. These features are clearly shown as hypointense areas. Therefore, we presume that a PTHR reflects peritumoral iron deposition.

Some degree of iron overload is commonly present in patients with chronic liver disease (CLD), such as alcoholic liver disease, nonalcoholic steatohepatitis, and hepatitis C [9]. T2*WIs are useful to identify HCC in CLD, and identifying HCC on T2*WIs is associated with differences in iron deposition in the HCC and liver parenchyma [10, 11]. Therefore, higher iron deposition levels in the background liver make it easier to identify HCC. A PTHR may also be influenced by iron deposition in the background liver.

Theoretically, iron deposition is involved in the presence of a PTHR; however, to the best of our knowledge, the association between a PTHR and the presence of histopathologic peritumoral iron deposition or iron deposition in the background liver has not been reported. Therefore, we aimed to investigate the PTHRs around HCCs on T2*WIs and the related histopathologic findings. We were especially interested in studying the association of PTHRs with peritumoral iron deposition or iron deposition in the background liver.

## Methods

### Patients

Our institutional review board approved this retrospective study and waived the requirement for written informed consent.

**Fig. 1:**
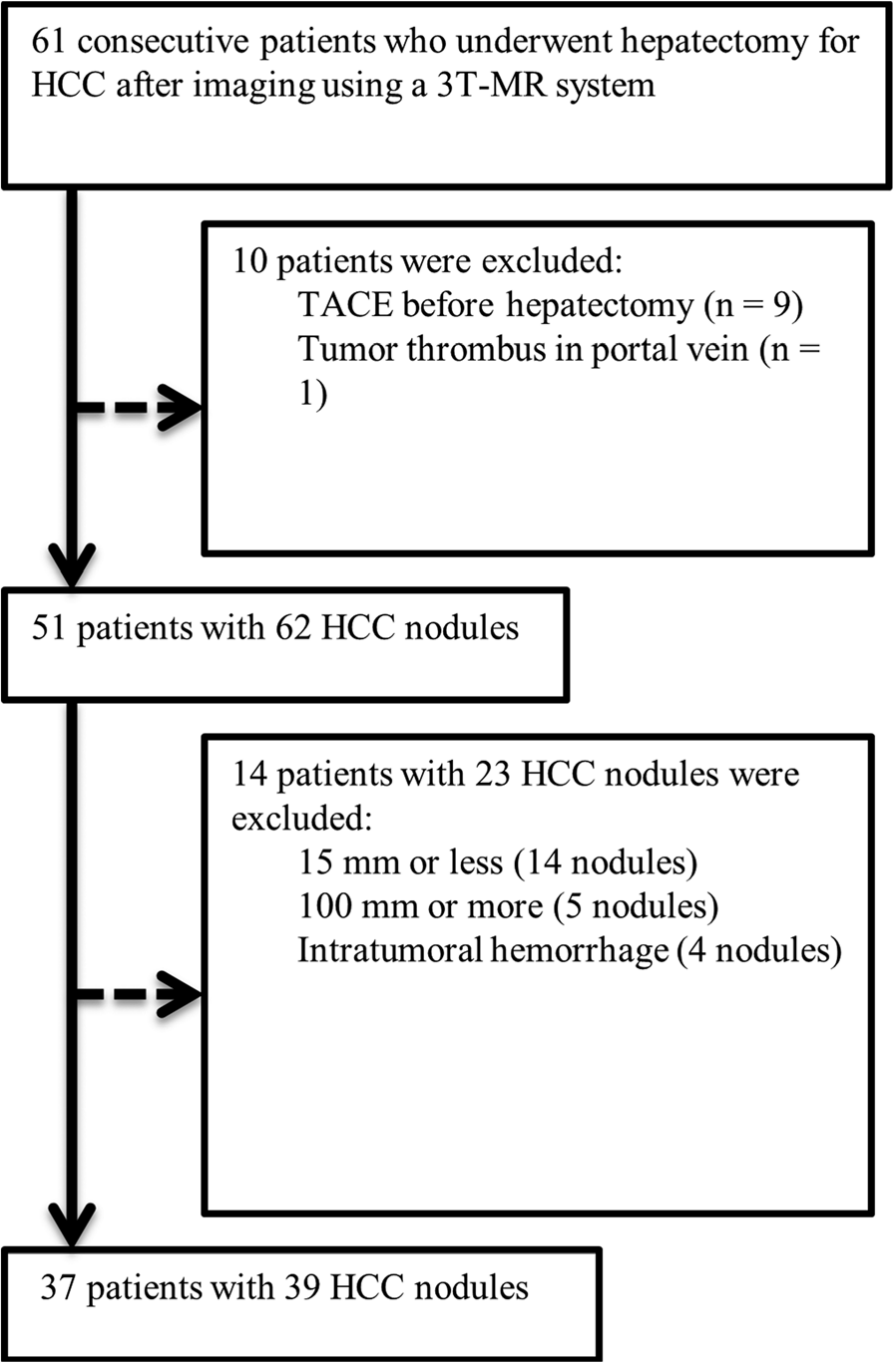
Flowchart showing the patient selection process in this study. *HCC* hepatocellular carcinoma, *MR* magnetic resonance, *TACE* transcatheter arterial chemoembolization

This study included consecutive patients who underwent hepatectomy for HCC after MR imaging from April 2012 through December 2013. The flowchart of the patient selection process is shown in Fig. 1. Choi et al. reported that HCCs smaller than 1.5 cm in size less frequently showed typical features on MR imaging than larger HCCs, and T2 hyperintensity, in particular, was not observed in small HCCs [12]. Therefore, we excluded HCCs < 1.5 cm. Large HCCs (> 10 cm) were excluded because the margin of these tumors often abutted the subcapsular region, making it difficult to analyze the peritumoral liver parenchyma.

During the study period, we evaluated 61 consecutive patients. Ultimately, 39 HCCs in 37 patients (25 males, 12 females; mean age, 69.3 ± 6.64 years) were selected. Tumor size was 1.5–4.8 cm (mean HCC size and standard deviation was 24.8 ± 8.40 mm). Twenty patients were infected with hepatitis C virus (HCV) (5 patients with hepatitis B virus (HBV)), and 12 patients did not have hepatitis B or C virus (alcoholic liver disease, eight patients; nonalcoholic steatohepatitis, three patients; unknown etiology, one patient). The grading of liver dysfunction was evaluated using the Child–Pugh classification; all patients had class A liver function. The interval between the date of MR imaging and hepatectomy ranged from 3 d to 58 days (median, 23 days). Progression-free survival (PFS) was calculated from the date of surgical resection to the date of imaging progression. Overall survival (OS) was calculated from the date of surgical resection to the date of death.

### MR imaging techniques

All MR images were obtained with a Magnetom Trio 3T MR system (Siemens Healthcare, Erlangen, Germany) using a six-channel body array coil and a six-channel spine matrix coil.

T2*WIs were obtained as part of the routine liver protocol in our institution. The sequence and scan parameters of the T2*WIs are shown in Table 1. We also obtained T1W1s (in-phase and opposed-phase), fat-suppressed T2WIs, diffusion-weighted images, and DCE-MR images routinely, and diagnosed the target nodule as HCC.

**Table 1:** Magnetic resonance sequence and scan parameters

	T2*WI
Pulse sequence	2D-GRE
TR (ms)	202–236
TE (ms)	7.38
Flip angle (°)	20
Matrix size	512 × 330–400
Acquisition time (s)	20–22
Field of view (mm)	340–420 × 223–315
Section thickness (mm)	4–5
Interslice gap (mm)	1

*2D*-*GRE* two-dimensional gradient-recalled echo, T2*WI T2*-weighted image, *TE* echo time, *TR* repetition time, *TSE* turbo spin echo

### Image analysis

**Fig. 2:**
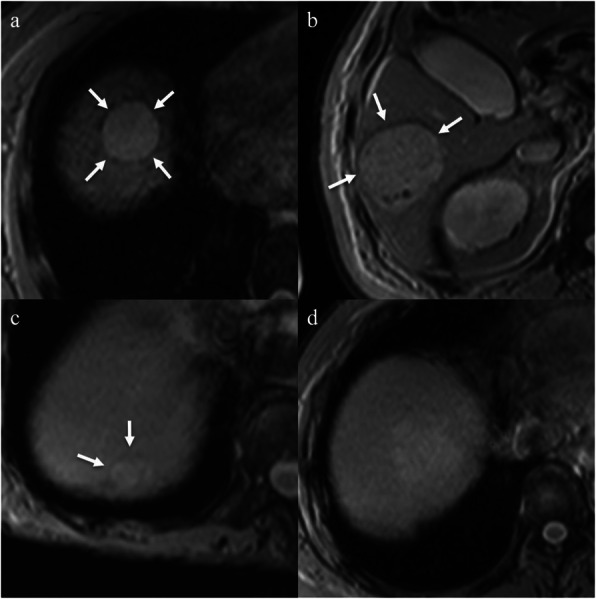
A peritumoral hypointense rim (PTHR) in T2*-weighted images in multiple select patients. **a** A PTHR surrounds the entire tumor (white arrows). **b** A PTHR is observed surrounding half of the tumor (white arrows). **c** A PTHR is observed surrounding one-third of the tumor (white arrows). **d** There is no PTHR around the tumor. The images in (**a**) through (**c**) are positive for a PTHR on T2*-weighted imaging. **d** The T2*-weighted image is negative for a PTHR

**Fig. 3:**
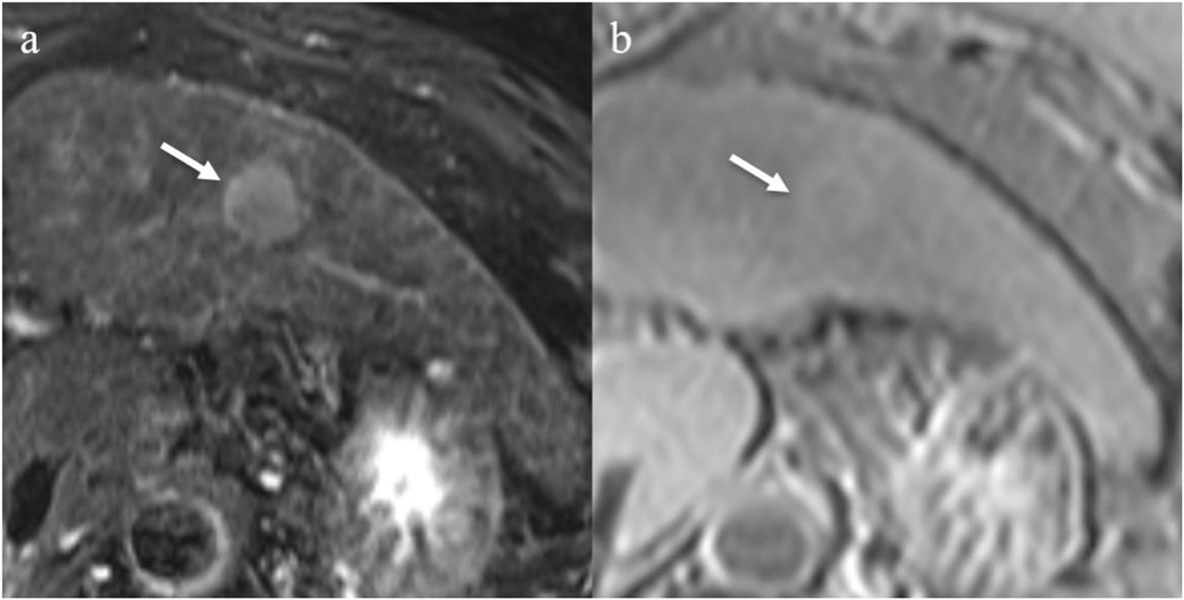
A 67-year-old female with hepatocellular carcinoma. **a** The tumor is seen as a hyperintense mass compared with the background liver on a T2-weighted imaging (white arrow). **b** The contrast between the tumor and the background liver is poor in the T2*-weighted image (white arrow)

We defined a PTHR as follows: low intense rim or band encompassing more than one-third of the tumor circumference on T2*WIs (Fig. 2). The purpose of this study was to compare T2*WIs and histopathologic features, but we reviewed T2*WIs compared with fat-suppressed T2-WIs because target nodules were sometimes difficult to detect (Fig. 3) and to distinguish the nodules from other lesions such as cysts on T2*WI alone [11]. Two readers (Y.T. with 11 years and Y.O. with 6 years of experience in abdominal imaging) confirmed the target nodule on T2WIs and then assessed the presence or absence of a PTHR on T2*WIs. Both readers were blinded to patients’ clinical information and histopathologic features, except for the diagnosis of HCC. Disagreement between the two readers was resolved by discussion and reaching consensus. All images were reviewed with EV Insite software (PSP Corp., Tokyo, Japan). The frequency of a PTHR was calculated after the assessment.

### Histopathologic analysis

In this study, we used Prussian blue staining to assess histopathological iron deposition in the liver. We used this method because there are numerous reports using Prussian blue staining as a histopathological evaluation method for iron deposition in the liver, and to our knowledge, there is no more accurate method of evaluating iron deposition. Slides of formalin-fixed, paraffin-embedded sections from resected liver specimens were stained with Prussian blue stain. Semiquantitative analysis was performed by one pathologist (T.U. with 20 years of experience in liver pathology), who was blinded to patients’ clinical information and imaging results, except for the diagnosis of HCC. One representative specimen per nodule containing the boundary between the tumor and the background liver parenchyma was analyzed while considering the MR findings because it was difficult to analyze the entire tumor circumference in large tumors. Iron deposition in the peritumoral liver parenchyma or background liver parenchyma (not adjacent to the tumor) was graded by the pathologist. The grade of iron deposition was as follows: grade 0, iron granules were absent or barely discernible in a high-power field (× 400 magnification); grade 1, granules were easily confirmed at × 400 magnification or barely discernible at × 250 magnification; grade 2, granules were resolved at × 100 magnification; grade 3, granules were resolved at × 25 magnification; and grade 4, masses were visible at low power (× 10 magnification) or with the naked eye [13, 14].

**Fig. 4:**
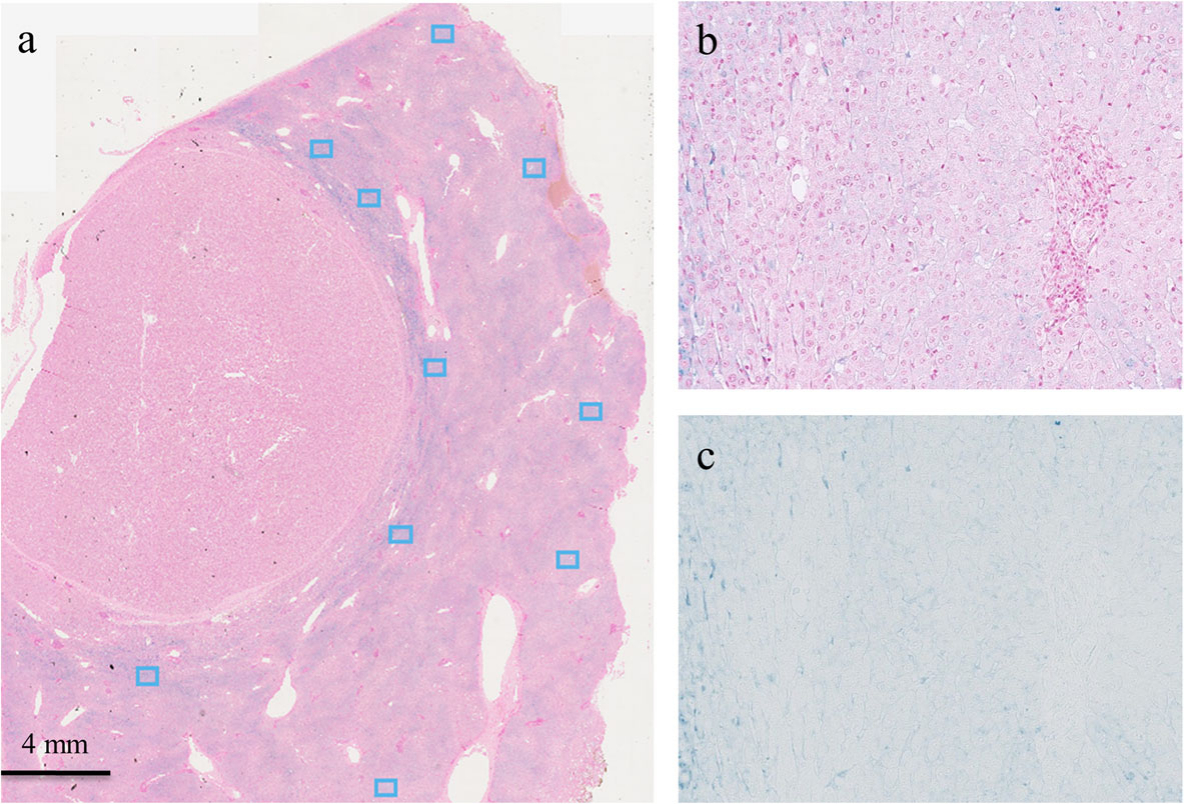
Microscopic slide image showing Prussian blue staining in a 76-year-old male with hepatocellular carcinoma. **a** A low-power field image. We randomly selected five individual fields (blue boxes) of peritumoral liver parenchyma or outer liver parenchyma for each tumor. **b**, **c** High-power multispectral images of the peritumoral liver parenchyma (× 200 magnification). Using a multispectral imaging system, we could extract only Prussian blue signals

Multispectral imaging can produce more accurate images than red, green, and blue (RGB) imaging because the wavelength range including visible light can be divided into a large number of channels. Therefore, we measured optical density using the Vectra 3 multispectral imaging system (PerkinElmer, Inc., Waltham, MA, USA). This quantitative analysis of iron deposition was performed on the same slide used in the semiquantitative analysis. Scan images of the whole specimen with Prussian blue staining were digitalized by the multispectral imaging system according to a previously published protocol [15]. Each of five individual fields (669 × 500 μm each) of peritumoral liver parenchyma or outer liver parenchyma for multispectral acquisition were selected randomly using Phenochart image viewer software, version. 1.0.4 (PerkinElmer, Inc.) to scan at high-power resolution (× 200 magnification) (Fig. 4). High-power multispectral acquisitions were analyzed using inForm image analysis software, version. 2.1 (PerkinElmer, Inc.) to extract the optical density of the Prussian blue staining. The mean optical density of five regions of peritumoral liver parenchyma or outer liver parenchyma was used for analysis.

Subsequently, we reviewed the pathology reports and investigated tumor grade, presence of a fibrous capsule, and degree of liver fibrosis. The degree of liver fibrosis in the background liver was graded according to the new Inuyama criteria as follows: no fibrosis (F0), portal fibrosis widening (F1), portal fibrosis widening with bridging fibrosis (F2), bridging fibrosis and lobular distortion (F3), or cirrhosis (F4) [16].

### Statistical analysis

All statistical analyses were performed using IBM SPSS Statistics for Windows, version 25.0 (IBM Corp., Armonk, NY, USA) and Bell Curve for Excel (Social Survey Research Information Co. Ltd., Tokyo, Japan). The Kolmogorov–Smirnov test was used to test for normality. Because of the non-normal distribution, the comparison between tumor size and etiology was analyzed using the Kruskal–Wallis test and Steel–Dwass test. Inter-reader agreement for the presence or absence of a PTHR on T2*WIs was assessed by calculating the kappa statistic [17]. A kappa value of ≤ 0.20 indicated poor agreement, 0.21–0.40 indicated fair agreement, 0.41–0.60 indicated moderate agreement, 0.61–0.80 indicated good agreement, and 0.81–1.00 indicated excellent agreement. The correlation between the semiquantitative and quantitative methods (peritumoral iron deposition and iron deposition in the background liver parenchyma, respectively) was analyzed using Spearman’s rank correlation coefficient. The association between PTHR and continuous variables in the potential contributing factors, such as peritumoral iron deposition (quantitative analysis), age, iron deposition in the background liver parenchyma (quantitative analysis), size, etiology, degree of fibrosis, and tumor grade, was analyzed using the Mann–Whitney *U* test. The association between the PTHR and binary variables in the potential contributing factors, such as sex and a histopathologic fibrous capsule, was analyzed using the *χ*^2^ test. The PFS and OS of the patients with and without a PTHR were analyzed by the Kaplan–Meier method and compared by the log-rank test. *P* < 0.05 was considered to indicate a statistically significant difference.

## Results

### Frequency of a PTHR on T2*WIs

On T2*WIs, a PTHR was observed in 23 of 39 HCCs (59%). The assessment of PTHR on T2*WIs differed between the two readers for 9 HCCs. With visual evaluation, the kappa value of the PTHR on T2*WIs was 0.5363, indicating moderate inter-reader agreement.

### Correlation between a PTHR and the histopathologic findings

A histopathologic fibrous capsule was observed in 25 of 39 HCCs. In the PTHR-positive group, 16 (64%) HCCs had a histopathologic fibrous capsule. In the PTHR-negative group, 9 (36%) HCCs had a histopathologic fibrous capsule. There was no significant difference in the histopathologic fibrous capsule between the PTHR-positive and -negative groups (*P* = 0.394).

**Fig. 5:**
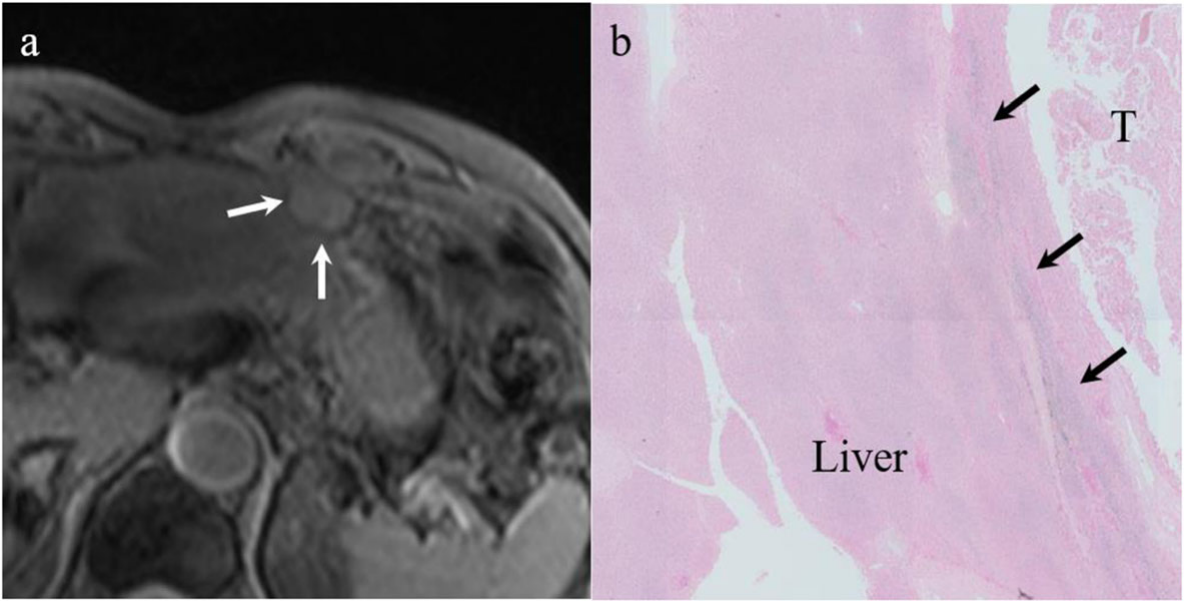
A 68-year-old male with hepatocellular carcinoma (HCC). **a** A T2*-weighted image shows the tumor with a peritumoral hypointense rim (white arrows). **b** This lesion was evaluated as grade 3 for peritumoral iron deposition (black arrows) and grade 1 for iron deposition in the background liver in a semiquantitative histopathological method. In addition, the mean optical density of this lesion was measured as 34,321.7 for peritumoral iron deposition and 19,475.5 for iron deposition in the background liver in a quantitative histopathological method. (Prussian blue stain; original magnification, × 20)

**Fig. 6:**
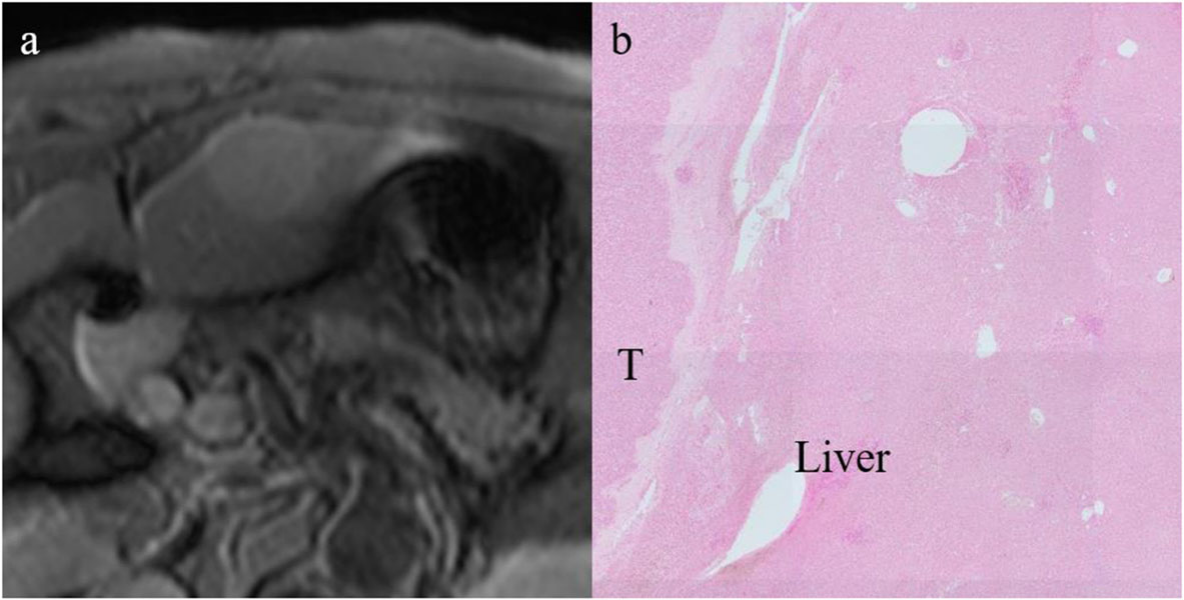
A 71-year-old male with hepatocellular carcinoma (HCC). **a** The T2*-weighted image shows the tumor without a peritumoral hypointense rim. **b** This lesion was evaluated as grade 0 for peritumoral iron deposition and grade 0 for iron deposition in the background liver in a semiquantitative method. In addition, the mean optical density of this lesion was measured as 6168.5 for peritumoral iron deposition and 5145.7 for iron deposition in the background liver in a quantitative histopathological method. (Prussian blue stain; original magnification, × 20)

In the semiquantitative histopathologic peritumoral iron deposition assessment, the 23 HCCs with a PTHR were classified as follows: grade 0 (*n* = 1); grade 1 (*n* = 2); grade 2 (*n* = 4); grade 3 (*n* = 6); or grade 4 (*n* = 10). In contrast, 16 HCCs without a PTHR were classified as follows: grade 0 (*n* = 12); grade 1 (*n* = 1); grade 2 (*n* = 1); grade 3 (*n* = 1); or grade 4 (*n* = 1). In the semiquantitative analysis, peritumoral iron deposition was significantly increased in HCCs with a PTHR compared with HCCs without a PTHR (*P* < 0.001). When evaluating the semiquantitative histopathologic iron deposition in the background liver, the 23 HCCs with a PTHR were classified as follows: grade 0 (*n* = 5); grade 1 (*n* = 2); grade 2 (*n* = 4); grade 3 (*n* = 9); or grade 4 (*n* = 3). The 16 HCCs without a PTHR were classified as follows: grade 0 (*n* = 14); grade 1 (*n* = 0); grade 2 (*n* = 1); grade 3 (*n* = 1); or grade 4 (*n* = 0). In the semiquantitative analysis, peritumoral iron deposition was significantly increased in HCCs with a PTHR compared with HCCs without a PTHR (Figs. 5 and 6) (*P* < 0.001).

**Fig. 7:**
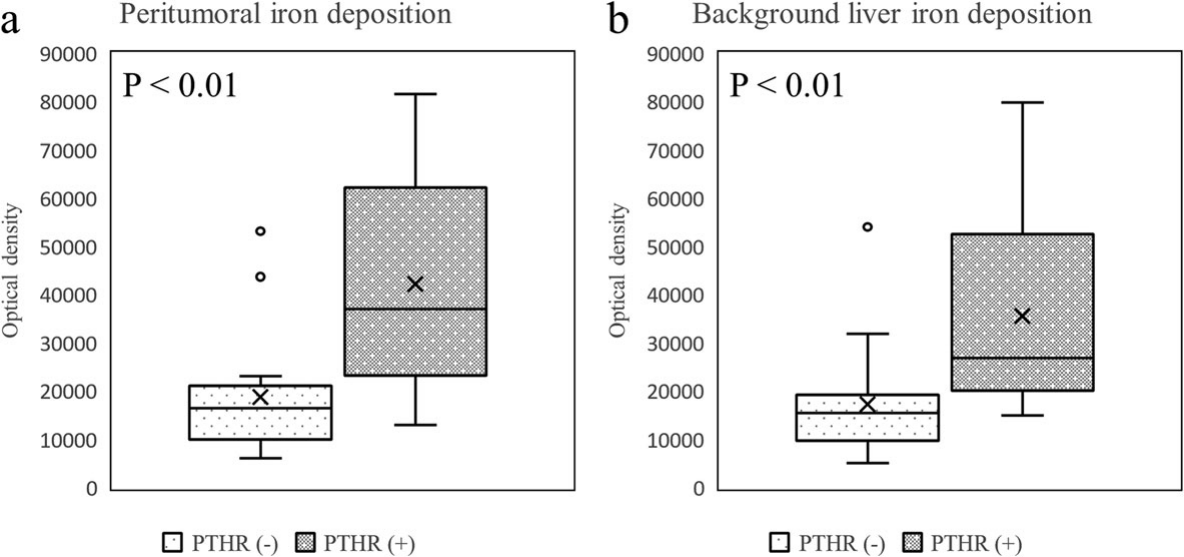
Results of comparisons between the presence of a PTHR and iron deposition. **a** PTHR presence compared with quantitatively assessed peritumoral iron deposition or **b** quantitatively assessed iron deposition in the background liver using the Mann–Whitney *U* test. There were significant differences in both cases (*P* < 0.001)

For the quantitative histopathologic peritumoral iron deposition, the mean optical density of HCCs with a PTHR was 42,244.1 ± 20,854.9, and that of HCCs without a PTHR was 18,739.1 ± 12,258.7. In the quantitative analysis of the histopathologic iron deposition in the background liver, the mean optical density of HCCs with a PTHR was 35,554.7 ± 19,854.8, and that of HCCs without a PTHR was 17,292.4 ± 11,605.8. In the quantitative methods, both peritumoral iron deposition and iron deposition in the background liver were significantly increased in HCCs with a PTHR compared with HCCs without a PTHR (Fig. 7) (*P* < 0.001).

The correlation coefficient of the peritumoral iron deposition between semiquantitative and quantitative methods was 0.705 (*P* < 0.001). The correlation coefficient for iron deposition in the background liver between the semiquantitative and quantitative methods was 0.729 (*P* < 0.001).

### Association between a PTHR and potential contributing factors

The difference between the presence of a PTHR and potential contributing factors, including clinical or histopathologic features, was also analyzed (Table 2). There were no significant differences in patients’ sex or age, or in the differentiation. However, there were significant differences in the size (*P* = 0.005), etiology (*P* = 0.001), degree of iron deposition in the background liver parenchyma (*P* < 0.001), and the degree of fibrosis (*P* = 0.042). The comparison between tumor size and etiology was analyzed using the Kruskal–Wallis test, and there was a significant difference (*P* = 0.021). Therefore, further analysis was performed using the Steel–Dwass method for multiple comparisons, and there was a significant difference between HCV (mean size: 21.7 ± 6.97 mm) and non-B, non-C (NBNC) (mean size: 29.8 ± 10 mm) (*P* = 0.02). Other comparisons showed no significant difference.

**Table 2:** Clinical and histopathological analysis among PTHR+ and PTHR− groups

Peritumoral hypointense rim	Positive	Negative	*P* value
Sex (M/F)	18/5	10/6	0.143
Mean age (years)	68.4 ± 7.34	70.7 ± 5.93	0.208
Mean size (mm)	26.7 ± 8.39	20.5 ± 6.91	0.005*
Etiology, HCV/HBV/NBNC	8/4/11^a^	14/1/1^b^	0.001*
Background liver fibrosis (F0/1/2/3/4)	1/4/7/5/6	0/1/2/5/8	0.042*
Differentiation (well/moderately/poorly differentiated)	6/16/1	5/9/2	0.455

F, degree of fibrosis using the new Inuyama classification (F0, no fibrosis; F1, portal fibrosis widening; F2, portal fibrosis widening with bridging fibrosis; F3, bridging fibrosis and lobular distortion; and F4, cirrhosis); *HBV* hepatitis B virus, *HCV* hepatitis C virus, *NBNC* (non-B, non-C). *Statistical significance

^a^HCCs with a PTHR were identified in 7 patients with alcoholic liver disease, 3 patients with nonalcoholic steatohepatitis, and one patient with an unknown etiology

^b^HCC without a PTHR was identified in one patient with alcoholic liver disease

### PFS and OS of patients with and without a PTHR

**Fig. 8:**
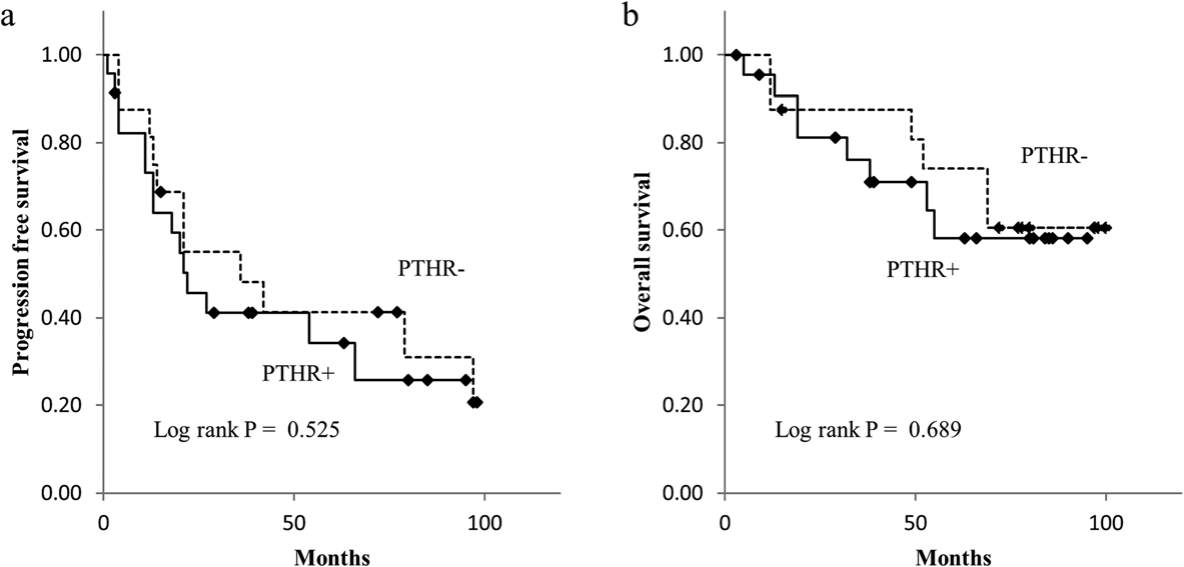
Kaplan–Meier curves. Curves are presented for progression-free survival (**a**) and overall survival (**b**), stratified by the presence of a PTHR

We analyzed the PFS and OS of the patients with and without a PTHR (Fig. 8). The mean PFS for the patients with and without a PTHR was 41.4 months and 49.6 months, respectively, and the mean OS for the patients with and without a PTHR was 68.4 months and 78.2 months, respectively. There were no significant differences in PFS and OS between the patients with and without a PTHR.

## Discussion

In this study, there was a significant, high association between peritumoral iron deposition and the presence of a PTHR compared with the absence of a PTHR. Interestingly, there was no significant association between a fibrous capsule and a PTHR. Therefore, we found that the presence of a PTHR on T2*WIs probably showed iron deposition in the peritumoral liver parenchyma. Furthermore, the presence of a PTHR was also associated with a significantly higher density of the background liver iron deposition.

As a more objective evaluation method, we performed a quantitative analysis of iron deposition as optimal density by multispectral imaging in addition to the semiquantitative method utilized in previous reports [12, 13]. Liu et al. studied the application of multispectral imaging in the quantitative immunohistochemical analysis of breast cancer. The authors reported that, in contrast to conventional RGB images, multispectral imaging was more accurate and reliable, and it provided more information on protein expression in relation to clinicopathological characteristics [18]. Multispectral imaging can be used for immunostaining as well as with other staining methods; therefore, this imaging method could be used without limitation with Prussian blue staining in this study of liver iron quantification. In normal color imaging (conventional RGB imaging), images are acquired as wavelength bands of light divided by the three RGB channels, whereas in multispectral imaging, images are acquired in more subdivided wavelength bands. Therefore, multispectral imaging can more accurately determine iron deposition with Prussian blue staining as the specific wavelength band. Because there was a significant correlation between the semiquantitative and quantitative methods, we analyzed the association between a PTHR and peritumoral iron deposition using the quantitative method.

Histopathologically, the capsule appearance on imaging features either a true capsule consisting of a fibrous inner layer and a prominent sinusoid outer layer, or a pseudocapsule consisting of a prominent sinusoid and/or peritumoral fibrous tissue [6, 19]. The presence of an enhancing capsule appearance is a characteristic feature of HCC, and this feature can suggest a favorable prognosis after hepatectomy and the degree of effectiveness of transcatheter arterial chemoembolization [20–22]. Therefore, assessing capsule appearance is important not only for a differential diagnosis but also for clinical management. This finding was adopted as a major feature in the LI-RADS [7]. In contrast, a nonenhanced capsule appearance, which is an ancillary feature favoring HCC in particular, can appear as a hypointense rim on T2WIs, nonenhanced T1WIs, or during the hepatobiliary phase of gadoxetic acid-enhanced MR images [2, 6, 23]. In particular, noncontrast MR images such as T2WIs and nonenhanced T1WIs are important for patients who cannot tolerate gadolinium, as a contrast agent (such as patients with renal failure and previous hypersensitivity reactions) [4, 5]. Chen et al. reported that T2*WIs and SWIs were superior to nonenhanced T1WIs and T2WIs for describing capsule appearance. The authors observed PTHRs in T2*WIs in 27 of 41 (66%) HCCs with the histopathologic capsule features, and PTHRs on SWIs were observed in 34 of 41 (83%) HCCs with the histopathologic capsule features. The authors speculated that an outer layer in the histopathologic capsule has abundant sinusoids with high concentrations of deoxyhemoglobin, resulting in a phase difference between the vessels and the surrounding parenchyma, although this finding has not been histopathologically analyzed in detail [8]. However, the finding may be useful for the diagnosis of HCC with a nonenhancing capsule appearance.

Although we did not histopathologically analyze the blood vessels of the capsule in this study, there was no significant difference in the histopathologic fibrous capsule between groups. However, the presence of a PTHR on T2*WIs was strongly associated with histopathologic peritumoral iron deposition. Therefore, we speculate that SWIs are more sensitive to change in magnetic susceptibility, such as with iron deposition, than T2*WIs, so a PTHR would be detected more clearly on SWIs than on T2*WIs. We attributed moderate inter-reader agreement (kappa value = 0.5363) to assessment of a PTHR on T2*WI because it was sometimes difficult to evaluate PTHR accurately owing to an insufficient signal–noise ratio. We also considered that better agreement could be obtained by SWI, which has better sensitivity to magnetic susceptibility and provides high spatial resolution.

A significantly higher degree of iron deposition in the background liver was detected in the presence of a PTHR compared with the absence of a PTHR. Hepatic iron deposition is commonly observed in CLD, and in particular, in alcoholic liver disease, nonalcoholic steatohepatitis, and hepatitis C [9]. In CLD, hepatic iron deposits are found in hepatocytes and reticuloendothelial system cells [24]. Furthermore, HCCs with a PTHR had a significantly higher density of the background liver iron deposition compared with HCCs without a PTHR. Therefore, we consider that the PTHR was easily observed around larger HCCs because of the compression change in the peritumoral liver parenchyma in the iron-deposited background liver.

There was a significant difference in the etiology of the HCCs, and the reason may be the significantly larger tumor size in NBNC patients compared with HCV patients. Moreover, HCCs with a PTHR were identified in 11 of 12 NBNC patients. In these 11 patients, 7 patients had alcoholic liver disease, 3 patients had nonalcoholic steatohepatitis, and one patient had an unknown etiology. Alcoholic liver disease causes hepatic iron overload from an early stage in approximately half of all patients, which is a higher degree of iron deposition than with viral liver disease [9]. Therefore, we considered that NBNC patients, who had a high incidence of alcoholic liver disease, also had high hepatic iron deposition and significantly more HCCs with a PTHR. Although hemochromatosis was not evaluated in our study, it is highly likely that HCC caused by hemochromatosis, in which iron is deposited in hepatocytes, will also show a PTHR.

There was significant difference in the degree of fibrosis between patients with and without a PTHR. Iron overload in the liver is associated with a high degree of fibrosis (F3 or F4) [9]. Iron deposition in the background liver was significantly increased in HCCs with a PTHR compared with HCCs without a PTHR, in this study. However, the degree of fibrosis was significantly lower in HCCs with a PTHR, which may be explained by the small sample size of our study population.

As we have mentioned, a PTHR can be considered to reflect peritumoral iron deposition, not a histopathologic fibrous capsule. Furthermore, a PTHR might correspond to the liver parenchyma with iron deposition compressed by the tumor. Iron deposition in CLD occurs mainly in Kupffer cells and hepatocytes. In this study, a PTHR was observed when there was a large amount of iron deposition in the background liver, and with large HCCs. Therefore, we considered that PTHR was not caused by microbleeding but instead, was caused by the increased iron density around the tumor owing to iron compression of the liver parenchyma by the tumor. Therefore, we presume that a PTHR can also be detected in other hepatic tumors if the tumors are larger, and if there is more iron deposition in the background liver. We consider that the future application of T2*WI depends on whether it can be useful in the diagnosis of HCC without using contrast agents. We also believe that the use of SWI can increase the detection rate of a PTHR. However, in the future, we need to study other hepatic tumors to determine whether the presence of a PTHR is a specific finding (i.e., a nonenhancing capsule appearance) for HCC.

There were no significant differences in PFS and OS between patients with and without a PTHR, and it was difficult to confirm the clinical usefulness of T2*WI in affecting the patients’ prognosis, in this study. Therefore, even if T2*WI is added as a sequence in MRI for suspected HCC, this addition does not affect the current treatment policy for HCC.

This study has several limitations. First, there was selection bias regarding the study population size because we excluded small HCCs (< 1.5 cm) and large HCCs (> 10 cm). Additionally, the start time was set for evaluation targets that were imaged using the 3T MR system. However, the evaluation time was short because of the time and costs involved in analyzing the targets with multispectral imaging. As a result, the number of HCCs was low. Second, the entire circumference of the tumor was not evaluated in the histopathologic examination.

## Conclusion

The presence of a PTHR in HCCs on T2*WIs was strongly associated with iron deposition in the peritumoral liver parenchyma but not with the fibrous capsule. The appearance of a PTHR might be affected by the degree of iron deposition in the background liver parenchyma and the tumor size.

## Data Availability

The datasets used and/or analyzed during the current study are available from the corresponding author on reasonable request.

## Abbreviations

PTHR: Peritumoral hypointense rim
HCC: Hepatocellular carcinoma
T2*WI: T2*-weighted image
MR: Magnetic resonance
DCE: Dynamic contrast-enhanced
T1WI: T1-weighted image
T2WI: T2-weighted image
LI-RADS: Liver imaging reporting and data system
SWI: Susceptibility-weighted image
CLD: Chronic liver disease
HCV: Hepatitis C virus
HBV: Hepatitis B virus
